# Soft and disordered hyperuniform elastic metamaterials for highly efficient vibration concentration

**DOI:** 10.1093/nsr/nwab133

**Published:** 2021-07-29

**Authors:** Hanchuan Tang, Zhuoqun Hao, Ying Liu, Ye Tian, Hao Niu, Jianfeng Zang

**Affiliations:** School of Optical and Electronic Information and Wuhan National Laboratory for Optoelectronics, Huazhong University of Science and Technology, Wuhan 430074, China; School of Optical and Electronic Information and Wuhan National Laboratory for Optoelectronics, Huazhong University of Science and Technology, Wuhan 430074, China; School of Optical and Electronic Information and Wuhan National Laboratory for Optoelectronics, Huazhong University of Science and Technology, Wuhan 430074, China; School of Optical and Electronic Information and Wuhan National Laboratory for Optoelectronics, Huazhong University of Science and Technology, Wuhan 430074, China; School of Optical and Electronic Information and Wuhan National Laboratory for Optoelectronics, Huazhong University of Science and Technology, Wuhan 430074, China; School of Optical and Electronic Information and Wuhan National Laboratory for Optoelectronics, Huazhong University of Science and Technology, Wuhan 430074, China; State Key Laboratory of Digital Manufacturing Equipment and Technology, Huazhong University of Science and Technology, Wuhan 430074, China

**Keywords:** elastic metamaterials, soft materials, disordered hyperuniformity, acoustic black holes, vibration concentration

## Abstract

Vibrations, which widely exist throughout the world, could be a nearly endless and locally obtained green energy source. It has been a long-standing challenge to efficiently utilize dispersed vibration energy, especially within the high-frequency range, since the amplitudes of high-frequency vibrations in local parts of objects are relatively weak. Here, for the first time, we propose a soft and disordered hyperuniform elastic metamaterial (DHEM), achieving a remarkable concentration of vibrations in broad frequency bands by a maximum enhancement factor of ∼4000 at 1930 Hz. The DHEM, with rational sizes from ∼1 cm to ∼1000 cm, covers a broad range of frequencies from ∼10 Hz to ∼10 kHz, which are emitted by many vibration sources including domestic appliances, factories and transportation systems, for example. Moreover, the performance of the soft DHEM under deformation is validated, enabling conformal attachments on uneven objects. Our findings lay the groundwork for reducing traditional energy consumption by recovering some of the energy dissipated by devices in the working world.

## Teaser

This paper presents a novel soft metamaterial design for concentrating vibration with an enhancement factor of ∼4000 by putting the disordered scatterers with a power-law curved edge in soft materials.

## INTRODUCTION

Vibrations widely exist in natural environments, living creatures and machines. Such vibrations, with different frequencies, could provide an accessible green energy source [1]. By collecting ubiquitous vibrations through micro-transducers such as piezoelectric nanogenerators [2–4] or triboelectric nanogenerators [5–7], it becomes possible to power wearable consumer electronics integrated in clothes, implantable devices in human bodies, portable terminals in the Internet of Things and even unsupervised vehicles in harsh environments. However, currently it is not practical to efficiently utilize the energy from dispersed vibrations, especially those within the high-frequency range (∼10^1^ to ∼10^3^ Hz), since the amplitudes of the high-frequency vibrations in local parts of objects are usually relatively weak. At the same time, the harvesting efficiency of micro-transducers is relatively low [8]. In addition, many potential vibration sources have soft bodies and/or uneven geometries. Thus, it is desirable to develop a flexible device to collect and concentrate vibrations over a broad frequency range.

Significant efforts have been invested in concentrating vibrations in rigid or soft materials by elastic artificial materials including phononic crystals (PCs) with point defects [9], graded refractive index materials [10], mechanical metamaterials [11,12] and triboelectric nanogenerators with structures [13,14]. Among these methods, the acoustic black hole (ABH) approach is an appealing way to dramatically concentrate vibrational energy [15–19]. An ABH design is a wedge-shaped structure with a decrease in thickness obeying the power-law profile. Theoretically, the group velocity of incident elastic waves will be reduced to zero when waves propagate to the tip of the ABH. At the same time, amplitude of vertical displacement will become infinite, resulting in non-reflection and extreme concentration of wave energy at the tip [15]. However, a truncated tip of ABH in practice does exist due to limitations in fabrication, thus greatly weakening the ABH performance and resulting in large reflecting coefficients (as large as 50%–70%) [15]. In addition, the rigid, irregular structure of traditional ABHs reduces the strength of the whole device and requires extra protection of the wedge tip.

Recently, the concept of hyperuniformity, which was first introduced to estimate point patterns according to their local density fluctuations, has attracted much attention [20,21]. Disordered hyperuniform (DH) structures suppress large-scale density fluctuations while they lack conventional long-range order. These structures were applied to classical wave systems recently to introduce large isotropic photonic/phononic band gaps (PBGs) that are capable of blocking light/sound in amorphous arrangements [22–24]. Thus, DH photonic/phononic structures (DHPSs) have been efficiently used for free-form waveguides [23,25], integrated waveguide polarizers [26,27], graded effective index materials [28,29], enhanced absorption [30,31], suppressing aliasing in images [32] and topological insulators [33]. However, few explorations of DH structures for applications in the elastic wave field have been reported.

Here, we report soft and DH elastic metamaterials (DHEMs) that can remarkably concentrate the energy of vibrations in broad frequency bands and eliminate reflections. The DH structure makes it possible to form a reflector with its edge obeying a smooth power-law profile, thereby resulting in high-efficiency ABH effects. In this situation, a remarkable concentration of vibrations in broad frequency bands by a maximum enhancement factor of ∼4000 at 1930 Hz could be achieved. Moreover, we tested the performance of DHEM under bending and compression conditions. The soft matrix enables the DHEM to conformally attach on uneven objects such as engines and transportation vehicles. Our findings also provide motivation to revisit many traditional wave-controlling structures.

## MECHANISM OF DHEM

**Figure 1.**
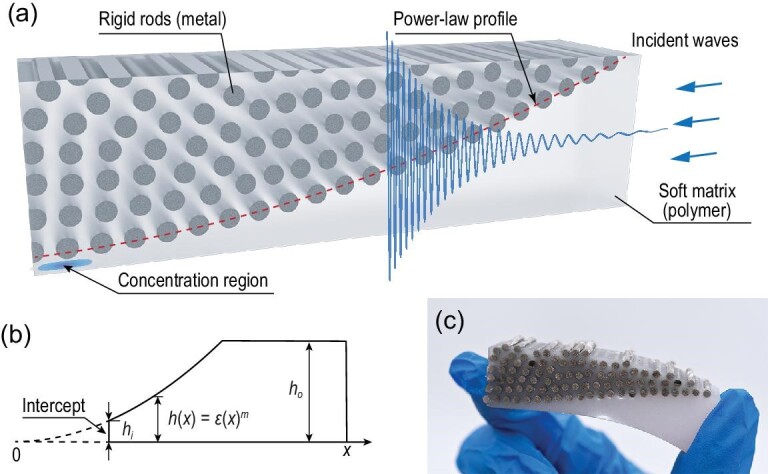
Vibration concentration by soft and disordered hyperuniform elastic metamaterials (DHEMs). (a) Schematic of a DHEM. When elastic waves are incident from the right side, most elastic energy will be concentrated at the ‘virtual tip’ (blue spot in Fig. 1a). (b) Sketch of traditional ABH. The main part is a boundary obeying a power-law profile $h = \varepsilon {x^m}(m \ge 2)$ with a bottom that is tangent to the boundary at the point *x* = 0, where *ϵ* is an arbitrary non-zero real number and *x* represents the bottom length of the wedge. But an intercept *h_i_* exists in the realistic production. (c) Photograph of a soft DHEM sample.

Figure 1a shows the DHEM design with optimized hyperuniform patterned rigid rods embedded in a soft polymer matrix. When the elastic waves, with their frequencies within the bandgap of a DHPS, come from one side, the smooth power-law profile edge of the DH pattern will provide a reflecting border, similar to the ideal ABH, concentrating the elastic energy at the ‘virtual tip’ of the DHEM. Moreover, the DHEM converts the power-law profile into an internal instead of an external border in the ABH case, allowing the DHEM to have an arbitrary external shape. For a traditional ABH design, only a boundary obeying a power-law profile with a bottom that is tangent to the boundary at one point is needed. But the wedge will have an intercept at the tip in production, dramatically weakening the concentration effect, as depicted in Fig. 1b. We conducted a simulation to illustrate this weakening effect in Fig. S1. Compared with a traditional ABH, it is easier to implement high-efficiency concentration with a DHEM since the thickness from the bottom to the profile is easier to control in manufacturing. In addition, the ‘edge’ formed by PBGs allows a larger interval between the bottom and the peripheral rigid rods. A soft matrix is used in DHEMs for flexibility, as shown in Fig. 1c.

**Figure 2.**
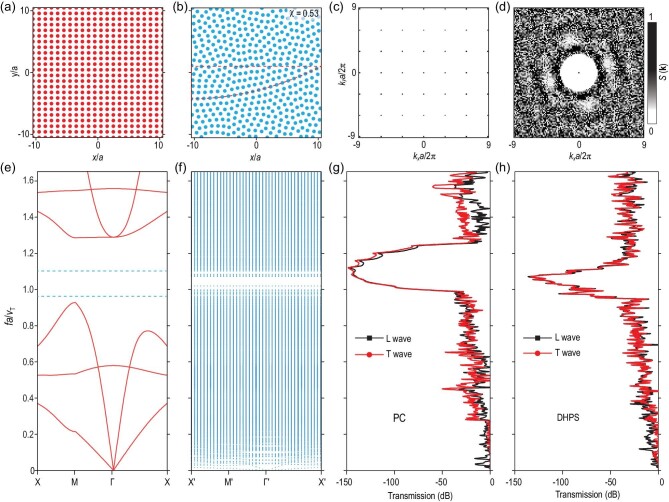
Characteristics of wave propagation in a phononic crystal (PC) and DHPS. (a) The periodic point pattern. (b) Original optimized DH point pattern with some points fixed to form a power-law profile. The parts of the DH pattern we utilized are highlighted with a red dash. (c and d) The structure factor *S*(**k**) for the periodic pattern and DH pattern in reciprocal space, respectively. The *S*(**k**) in (c) show Bragg peaks. The *S*(**k**) in (d) was optimized to be zero when ${|}$**k**${|}$ ≤ *K*, indicating hyperuniformity in the pattern. (e) Band structure of the periodic structure. (f) Band structure of the DHPS (folded in the supercell). (g and h) Transmission through a slab of PC and DHPS that is finite along the x direction (length $\sqrt N a$), respectively. Transmission is shown for both longitudinal (L wave) and the transverse (T wave) polarizations.

We optimized a DH scatter pattern first in order to design the actual DHEM. The entire DH scatter pattern with 441 points was optimized to be hyperuniform by fixing some starting points (see Supplementary Data). Figure 2a and b illustrates a periodic scatter pattern and the optimized DH scatter pattern, respectively. The criterion of hyperuniformity was defined by structural factor *S*(**k**) of point patterns. For an *N* points pattern, *S*(**k**) is defined as in Refs [20,21]:

(1) \begin{equation*} S( {{\bf k}}) = \frac{1}{N}\left| {\sum\limits_{n = 1}^N {{e^{i{{\bf k}} \cdot {{{\bf r}}_n}}}} } \right|, \end{equation*}

where **k** are wave vectors in the reciprocal space and ${{{\bf r}}_n}( {n = 1, \ldots ,N} )$ are the position vectors of the points. If *S*(**k**) satisfy $\mathop {\lim }_{{{\bf k}} \to 0} S( {{\bf k}} ) = 0$, the point pattern is hyperuniform. For a periodic case that obeys the criterion for hyperuniformity, the *S*(**k**) distribution in **k**-space is largely blank with periodic Bragg scattering peaks (Fig. 2c). For the DH case, as depicted in Fig. 2c, *S*(**k**) is nearly zero in a certain radius *K* of wave vectors. The radius *K* can influence the degree of ordering *χ* of the DH pattern. Low *χ* or a highly disordered state will lead to some localization effects [34–36]. The χ parameter is set to be 0.53 in the optimization process, corresponding to the existence of band gaps. Therefore, both the periodic and disordered structures are hyperuniform but the DH structure has an isotropic response and disordered point distribution.

To further characterize the hyperuniformity, we studied the dispersion relation of both the square-lattice PC and DHPS. We supposed that the phononic configurations were composed of two materials—soft polymer (Ecoflex^TM^-0030) as matrix and rigid rods (stainless steel) as fillers. In our

configuration, the lattice constant for PC (average interval for DHPS) was set to be 2.9541 mm and the filling ratio (by area) was 0.36. Thus, the diameter of rigid rods was 2 mm. The density${\rho _{rods}}$, Young’s modulus ${E_{rods}}$ and Poisson's ratio ${\upsilon _{rods}}$ of rigid steel rods were 7900 kg/m^3^, 195 GPa and 0.247, respectively. We experimentally measured the strain-stress relation of the soft matrix, as shown in Fig. S2. As our experiments were conducted in the small strain regime, we fitted the linear region to obtain the Young's modulus of the matrix; we used the following simulation parameters: the matrix density ${\rho _{matrix}}$, Young's modulus ${E_{matrix}}$ and Poisson's ratio ${\upsilon _{matrix}}$ of matrix are 1070 kg/m^3^, 100 kPa and 0.49, respectively. We calculated the velocity of transverse wave ${v_T}$ to be 5.544 m/s using ${v_T} = \sqrt {\frac{{{E_{matrix}}}}{{2{\rho _{matrix}}( {1 + {\upsilon _{matrix}}} )}}} $. We obtained the dispersion relations of both the PC and DHPS through a finite element analysis software (COMSOL Multiphysics). In such a square-lattice PC, Fig. 2e shows that there is a complete PBG with a normalized frequency ranging from 0.93 to 1.28 due to the Bragg scattering. In terms of actual frequencies, the bandgap ranges from 1745 Hz to 2400 Hz. For the DH case, we constructed a supercell with 441 points and calculated its dispersion relation. Bandgaps at similar positions were noted, as shown in Fig. 2f. Specifically, the frequency bandgap ranges from 1800 Hz to 2000 Hz. Moreover, transmissions of both longitudinal waves and transverse waves of PC and DHPS further indicate the existence of a PBG, as shown in Fig. 2g and h, respectively. The models for calculating dispersion relationship and transmission are given in Figs S3 and S4, respectively. The filling ratio also influences the dispersion relationship of the DHPS, as shown in Fig. S5.

## EFFECTS OF DHEM

**Figure 3.**
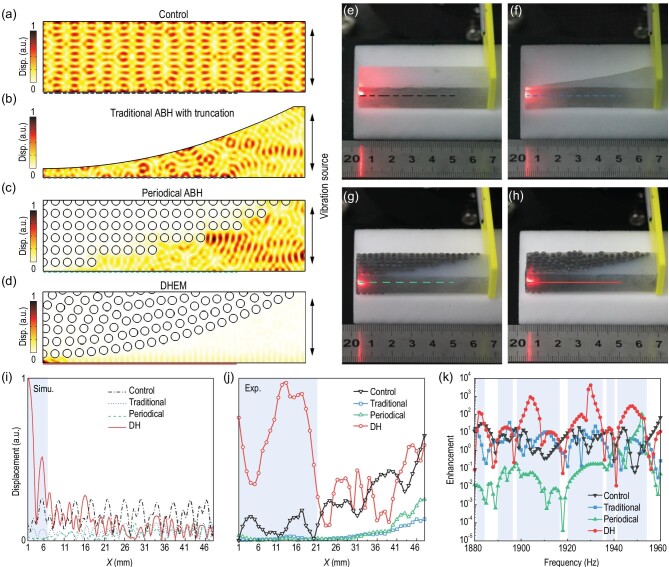
Characterization of the concentration of vibrational energy for various structures. (a–d) Simulated total displacement distribution when exciting elastic waves at 1930 Hz from the right side of four models. Panels top to bottom: (a) control, (b) traditional ABH, (c) periodic ABH and (d) DHEM. (e–h) Experimental samples of models: (e) control, (f) traditional ABH, (g) periodic ABH and (h) DHEM. The samples were sprayed with reflective spray at the front sides to enhance the reflection of incident laser. (i) Simulated displacement profiles along the bases of the four models where *x *= 0 corresponds to the left edge of the structures shown in (a–d). (j) Measured displacements on the lines indicated in (e–h). (k) Simulated enhancements for the four structures over the 1880–1960 Hz region of the spectrum. The enhanced regions are highlighted with a light blue color.

We constructed the DHEM by extracting part of the scattering from the entire pattern with 441 points (Fig. 2b) along a power-law edge profile (function: $y = {\rm{0}}{\rm{.25}}{x^2}$). This extraction is reasonable because parts of a DH distribution are also hyperuniform [21]. To make a DHEM, we arranged rigid rods in the soft matrix according to the distribution of the extracted DH pattern. In order to illustrate the effect of the DHEM, we assumed elastic waves propagating through four kinds of configurations and conducted two-dimensional simulations and experiments (Fig. 3).

Figure 3a–d depicts the simulated distributions of vibrations in a control group (a uniform polymer matrix), a traditional ABH with an interception at the tip, a periodical ABH (extraction of a stepped power-law profile edge from the periodic pattern) and a DHEM when elastic waves at 1930 Hz are incident from the right side, respectively. In the control group, elastic waves are reflected and form a standing wave (Fig. 3a). In the traditional ABH (Fig. 3b) and the periodic ABH (Fig. 3c), most elastic waves are reflected and localized to different regions due to the presence of the truncated tip (traditional ABH) and the serrated edge (periodic ABH). In the DHEM, most elastic waves are highly concentrated at the virtual tip, with few reflecting waves (Fig. 3d). To estimate the degree of concentration of vibrational energy, we defined enhancement as the ratio of the total elastic energy density in two different regions, as shown in Fig. S6. The simulated enhancements for the configurations shown in Fig. 3a–d are 1.57, 4.80, 0.18 and 4228.60, respectively. For comparison, previously reported enhancements are ∼100 to the best of our knowledge [8–13].

Moreover, by analyzing the vibrations on the bases of four models (outlined in Fig. 3a–d), the vibration intensity of the DHEM is obviously higher than those of the other configurations, as depicted in Fig. 3i. The total vibration intensity near the tips can be calculated by integrating the profiles in the light blue background regions; the total vibration intensities near the tips of the control, traditional and periodic cases are 31.97%, 11.56% and 1.32% of that of the DH case (100%), respectively. As shown in Fig. S4e–h, the concentration effect is pronounced for both the longitudinal (L) wave and transverse (T) wave.

We made the four simulated configurations into realistic samples with a height of 2 cm and measured their performance using the experimental set-up shown in Fig. S7. We utilized a scanning laser Doppler vibrometer (LDV) to measure the side displacement, referred to as the shear wave incident from the right side that is produced with an exciter. We scanned the vibration at the side (as shown by the photographs in Fig. 3e–h) and the experimental results are illustrated in Fig. 3j. By comparing different profiles, we found that the displacements of the DHEM were much higher than those of the others, which is consistent with the simulation results in Fig. 3i. The measured total vibration intensities near the tips of the control, traditional and periodic cases are 13.20%, 1.87% and 1.43% of the DH case (100%), respectively. By analyzing each individual profile, the displacements were found to decay when the *x* decreased toward the tip in all four cases. We attribute this decay to the intrinsic damping of soft polymers but the vibrations are still highly enhanced at the tip in the DHEM case when compared to other configurations.

We only demonstrated the experiments at a frequency of 1930 Hz in Fig. 3j, but the DHEM indeed fits for a broad frequency range within the bandgap of the DHPS. As shown in Fig. 3k*,* we conducted simulations to describe the vibration concentration by the DHEM over a range of frequencies; these simulations show that the elastic waves are dramatically enhanced across the bandgap in several frequency regions with the enhancement in the DHEM case being higher than that of the control case at more than 80% frequencies in the 1880–1960 Hz range. This frequency range could be extended by scaling the sample size or changing the matrix material (e.g. Ecoflex-0010 with lower modulus).

The thickness of the virtual tip, which is the distance between the bottom to center of the lowest rod, is critical to the concentration effect of the DHEM and we conducted a simulation to optimize the thickness of the virtual tip in the DHEM (Fig. S8). Compared to traditional ABHs, the DHEM allows a larger thickness of the virtual tip, making fabrication easier. Furthermore, as shown in Fig. S9, most elastic waves still concentrate at the target area even without the reflective border at the virtual tip. In addition, the power-law profile of the interface (see Fig. 1a) can be steeper or flatter. We constructed a DHEM with a steeper power-law profile interface that is defined by $y = 4{x^2}$, as depicted in Fig. S10a–c. In the DHEM case, the power-law profile becomes the internal interface, which means a nearly arbitrary upper boundary can be applied. Using simulation, we further demonstrated that a

DHEM with an irregular upper boundary also exhibits good concentration effects (Fig. S10d). Thus, DHEMs possess strong adaptability and flexibility in design.

## PERFORMANCE OF DHEM UNDER DEFORMATION

**Figure 4.**
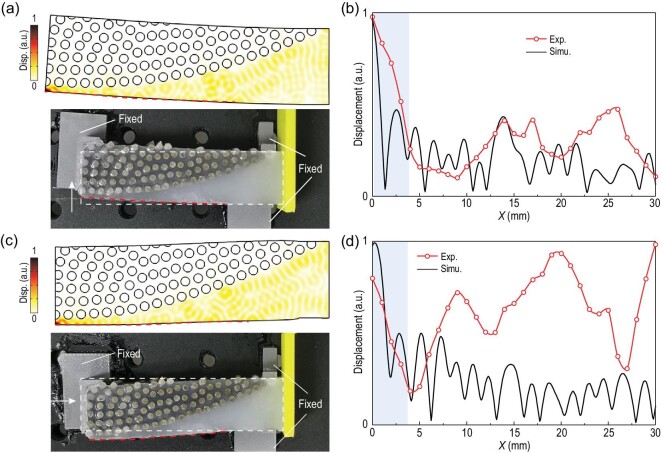
Performance of the DHEM under deformation. (a) Top: simulated total displacement distribution under bending when exciting elastic waves at 1930 Hz are incident from the right side. Bottom: photograph of a DHEM sample that is bent toward the top. White dashed lines indicate the original shape. (b) Simulated and experimental displacements on the red dashed lines in (a). (c) Top: simulated total displacement distribution under compression when exciting elastic waves at 1930 Hz are incident from the right side. Bottom: photograph of a DHEM sample that is compressed on the left side. White dashed lines indicate the original shape. (d) Simulated and experimental displacements on the red dashed lines in (c).

The soft matrix allows a DHEM to be applied to objects with arbitrary surface topography and we studied the performance of DHEM under deformation (Fig. 4). We examined typical bending and compression deformations as shown in Fig. S11. While the soft DHEM works well under small deformations, large deformations cause failure in the concentration effects due to a drastic change of the power-law interfacial profile and the DH lattice (Fig. S12). The upper panels of Fig. 4a and c depict the simulation of DHEMs under bending (pulled up one side by 3 mm) and compression (compressed by 2 mm), respectively. The simulations show that the DHEMs are able to concentrate vibrations under either bending or compression. The bottom panels of Fig. 4a and c show the photographs of DHEM samples. For bending, experimental measurement of the vibration concentrating effect near the tip was roughly consistent with the simulations (Fig. 4b). However, in the compression case, the experimental results show a concentration effect at the tip as well as in some other parts of the structure, which we attribute to a change in the effective modulus and density of the soft matrix under compression. Generally, the soft DHEM works well to concentrate vibrations under relatively small deformation meaning that DHEM should be suitable for many application scenarios.

## CONCLUSION

The essence of DHEMs is to replace the wedge structure of ABHs with DHPSs in order to reduce the difficulty of fabrication and to provide a realization of the theoretical ABH. The simulation results show that the DHEM allows a little manufacturing deviation, as shown in Figs S13 and S14. Moreover, the DHEM makes the power-law profile an internal instead of an external border in the ABH case, enabling the DHEM to have an arbitrary external shape (e.g. a rectangle with better structural stability instead of a wedge with a fragile tip). Furthermore, our method provides a platform to integrate more functional structures like waveguides and graded metamaterials with DH patterns.

It should be noted that the bandwidth of the DHEM can be further controlled by changing the filling ratio of the phononic structure, as shown in Fig. S5. This allows tailoring the dispersion relationship in a DHPS so that a narrower or wider bandgap can be achieved. In addition, the working frequency of a DHEM can be shifted to lower or higher bands by changing the sample sizes or material parameters of the matrix and fillers. Thus, the working frequencies of a DHEM extend from ∼10 Hz to ∼10 kHz with sizes in the range of ∼1 cm to ∼1000 cm; this covers the noise spectrum for most domestic appliances, factories and transport vehicles. We constructed four DHEMs whose working frequencies are located between 60 Hz and 50 000 Hz by simulations, as illustrated in Fig. S15. Enhancement factors of these DHEMs are over 5000. Additionally, we summarized and compared various mechanisms of vibration harvesting in Table S1. Our method performs best in enhancement, working frequency range, deformability and size.

In summary, by using a DH design, for the first time, we developed a novel ABH device that is easily fabricated with low cost while functioning as a robust and efficient vibration concentrator that can be attached to objects with uneven geometries. This will lead to new applications that improve many traditional wave-controlling structures. Additionally, by constructing these soft DHEMs, we have successfully overcome existing challenges in weak vibration sensing and considerably improved the concentration of vibrational energy. Thus, our study lays the groundwork for fully utilizing the vibration energy that is widely distributed in nature and provides a promising way to enhance vibration sensing in industry, reduce traditional energy consumption, charge long-term working devices in the Internet of Things or unmanned detection vehicles, and, as a result, build a greener world.

## METHODS

### Optimization of DH pattern

The DH patterns were obtained with a constrained optimization method. The procedure was carried out using, namely, the ‘collective coordinate’ method [21]. More details are shown in the supplementary data.

### Numerical calculation

COMSOL Multiphysics 4.2a was used to conduct the finite element analysis for simulating the dispersion relationship of phononic configurations, elastic waves propagating in soft matrix, and the structure deformation of DHEMs under strain. Low strain and low linear deformation were also ensured in most simulation cases. The type of the mesh element is free triangular and the maximum size is 0.3 mm (∼$\lambda$/10) in the simulation of elastic waves concentration. A Neo-Hookean hyperelastic model is utilized to simulate large structure deformation of the DHEM. Related parameters are shown in the supplementary data.

### Preparation of experimental samples

Molds with designed holes in the base were printed by a three-dimensional printer (A8, JGAURORA Co.). Then the rigid rods (304 stainless steel) were inserted into the molds to form specific distributions. The matrix materials (Ecoflex-0030 or Ecoflex-0010) were poured into the molds. After being placed at 60°C for 4 hours, the samples were formed and taken out. More details are shown in the supplementary data.

### Measurement set-up

The testing platform was constructed with an optical bench, acoustic sponges and a vibration source, as depicted in Fig. S6. A scanning LDV (LV-S01, Sunny Optical Technology Co. Ltd) was used to measure the displacements of samples.

## Supplementary Material

nwab133_Supplemental_File
