# Supplementary material for: Soft and disordered hyperuniform elastic metamaterials for highly efficient vibration concentration
Source: Natl Sci Rev. 2021 Jul 29;9(1):nwab133. doi: 10.1093/nsr/nwab133 (PMC8783669; doi:10.1093/nsr/nwab133)
Supplement: nwab133_Supplemental_File [file nwab133_supplemental_file.docx]

Supplementary data for

**Soft and Disordered Hyperuniform Elastic Metamaterials for Highly Efficient Vibration Concentration**

Hanchuan Tang, Zhuoqun Hao, Ying Liu, Ye Tian, Hao Niu, Jianfeng Zang

* E-mail: [jfzang@hust.edu.cn](mailto:jfzang@hust.edu.cn) (J.Z.)

**This PDF file includes:**

Supplementary text

Figures S1 to S15

Table S1

Supplementary Information Text

**Optimization of disordered hyperuniform pattern.** The disordered hyperuniform (DH) patterns were obtained with a constrained optimization method. The procedure was carried out using namely “collective coordinate” method [21]. Briefly, the structure factor *S*(**k**), defined as $1+\frac{2}{N}\sum_{i,j} \cos\left[\mathbf{k}\left(\mathbf{r}_{i}-\mathbf{r}_{i}\right)\right]$, was minimized to nearly zero in the k-space within radius K, where N is the number of cylinders, **r**_i_ and **r**_j_ are the position of cylinders centers in the patterns, and *k* is the wave vector. To drive the minimization process, we used the Quasi-Newton algorithm for optimization. The stealthy parameter defined by $\chi=\frac{M\left(\mathbf{k}\right)}{N}$ reflects the degree of uniformity, where *M*(**k**) is the number of linearly independent **k** vectors in the radius *K*. For example, when *N* = 418, the pattern is disordered when *χ* < 0.58 and is crystalline when *χ* > 0.78*.* Disordered hyperuniform acoustic black hole (DHEM) is designed based on DH patterns. We acquired DHEM pattern by constraining some start points [25]. First, 22 evenly distributed cylinders are placed and fixed on a designated path as an “edge”. Here the path was defined as$f\left(x\right)=0.25x^{2}$. Other cylinders were randomly sat within a square box. Then these initially random cylinders were optimized into a hyperuniform pattern. As a result, redistributed cylinders were settled around the parabolic “edge”. At last, the wanted part of the pattern (edge included) remains and the rest will be removed. As shown in Fig. S8, the remaining part itself could also be seen as a DH pattern. In our case the stealthy parameter *χ* was set to 0.53.

**Suboptimal effect in traditional acoustic black holes (ABH).** Fig. S1 shows an ideal ABH and two types of suboptimal practical ABHs. Fig. S1 b shows a case where a cut-off presented at the edge, due to the limitation of fabrication. Fig. S1c depicts an alternative method to prepare the ABH by thickening the bottom. Compared to the ideal case on the right column, suboptimizations cause obvious reflections at the intercepted end, resulting in a low efficiency of ABH in practical applications.

**Materials and experiments.** We examined many material configurations in order to balance the enhancement, working frequency band, flexibility of device, and sample size. Finally, we chose steel rods as the fillers and Ecoflex^TM^ as matrix. Before being used for the disordered hyperuniform elastic metamaterials (DHEM) construction, several types of Ecoflex were examined by uniaxial tensile tests. First, two flat specimens with shoulders (used with serrated grips) were prepared, using Ecoflex-0010 and Ecoflex-0030 (Fig. S2a). As shown in Fig. S2b, the data within the “small deform region” were picked out and used to establish a fitting profile. The slopes of fitting curves show Young’s modulus of standard samples. Neo-Hookean hyperelastic model is utilized to simulated large structure deformation of DHEM. Related mechanical parameters *μ*, i.e. initial shear modulus is 32.8 kPa, which is fitted from Fig. S2b. To carry on the experiments in Maintext Fig. 3, we first constructed the model molds with customized holes by additive manufacturing. Then steel rods were inserted into the holes to form specialized pattern. Next, the soft host material was filled into the mold. After the host was cured sufficiently, the samples with steel rods were taken out. The testing platform was constructed with an optical bench, acoustic sponges, a vibration source, and a scanning laser Doppler vibrometer (LDV), as depicted in Fig. S7. Figure S11 illustrates how to enforce a certain deformation to our DHEM sample. For experiments, three fastener parts were made by 3D printing. Then they were stuck to the samples where the positions as defined in the Fig. S11a. Two clamps on the right function as fixed constraints while the L shape rigid connector on the left is used to perform rigid connector. When three constraint parts are in position, they would be stuck to the platform, and then the setup is ready for experiments.

**Band structure of PC and DHPS.** In our numerical simulations and experiments, structural steels and Ecoflex^TM^ were utilized as fillers and host, respectively. The dispersion relationships were calculated with FEA software COMSOL Multiphysics 4.3a. For calculating dispersion relationship of DHEM, a square with 441 points arranged according to DH distribution is considered as a single cell (which is called “super cell”), as shown in Fig. S3. The four borders of the whole square are set to be Floquet periodical boundary. For numerical simulation of transmission, as shown in Fig. S4, the patterns were defined within a two-dimensional square box, with free boundary conditions on the top and bottom edge. The structure was subjected to a vibration exciter on the left edge. The displacement data were collected by integrating over the “incident” region (at the vibration source) and “dissipating” region (at the low-reflection boundaries). At the same time, filling ratio (calculated by area) will influence the properties of DHPS, as shown in Fig. S5. As the filling ratio increases, the width and central frequency of band gap will increase either. When filling ratio is too low (for example ~0.04), bandgaps almost vanish.

**Optimization for DHEM design.** Besides the DHEM patterns itself, the container that wraps the whole structure should be delicately designed. The container is made of the same material as the host. Here two important parameters will be discussed. We defined the vertical distance between the center of left bottom cylinder to the bottom as *d*, as well as the width of container as *h*, as shown in Fig. S8. We numerically simulated the performance of the DHEM with different parameters. The results are shown in Fig. S8c and D, where the enhancement is defined by the vibration energy density of region 1 over region 2. The criteria for good performance are high enhancement and wide working bandwidth. According to these, several configurations perform similarly and the difference between those matters little to the big picture. Finally, we chose the optimal parameters to construct the DHEM container.

**The universality of the designing method.** A mirror model to verify the design of DHEM is depicted in Fig. S9. The numerical evaluation of a steeper edge version of DHEM was also conducted, as shown in Fig. S10a-c, where the edge profile was defined by$f\left(x\right)=2x^{2}$. The result shows that our method could be applied to different types of edge-profiles. Moreover, in Fig. S10d the DHEM with an amorphous upper border is presented. The enhancement of elastic energy is still obvious in this structure. It can be concluded that as long as the edge and some of the inner cylinders (cylinders above the edges, in our graph) persevered, the function of DHEM remains. Above all, our designing method meets various applicable situations through slight adaption to the required scenario. More DHEMs with the working frequencies between 60 Hz and 50000 Hz are designed, as illustrated in Fig. S15. The Young’s modulus (used in simulation) of Ecoflex-0010, Ecoflex-0030, and Polydimethylsiloxane (PDMS) are 30 kPa, 100 kPa, and 1000 kPa, respectively. The density (used in simulation) of Ecoflex-0010, Ecoflex-0030, and Polydimethylsiloxane (PDMS) are 1070 g/cm^3^.

**The influence of position deviation.** The position deviation of every scatterer will increase the sum of structure factors in a certain radius of wave numbers. But there is no need to optimize the structure factor to be absolutely ‘zero’. In our case, the sum of structure factors in a certain radius of wave numbers is about 10^-2^. We optimized other DH patterns with the sum of structure factors to be 10. These DHPS exist bandgaps either. Besides, the performance of DHEM under deformation indicate the DH distribution can resist the negative impact from slight position changes of scatterers, as shown in Maintext Fig.4. We also added a simulation to further clarify the influence of position deviation (may come from deformation or manufacturing deviation) in Fig. S13. The effective lattice constant *a*, i.e. average distance between the scatterers, is 2.9541 mm. We give every scatterers an extra random position deviation Δ*P*. In order to prevent the collision of scatterers, the maximum Δ*P* is set to be 0.15*a.* Enhancement factors for DHEM with different Δ*P* in broad frequencies (1880 Hz to 1960 Hz) are shown in Fig. S14. The DHEM maintain fairish concentration effects when Δ*P* < 0.1*a* (optimum working frequency may slightly change). The scatterers show somewhat Anderson Localization until Δ*P* increases to ~0.15a. Therefore, we think DHEM allows a little manufacturing deviation.

**Summary and comparison of various mechanism of vibration harvesting.** We summarized and compared various mechanism of vibration harvesting in Table. S1. Our method performs best in enhancement, working frequency range, deformability, and size.


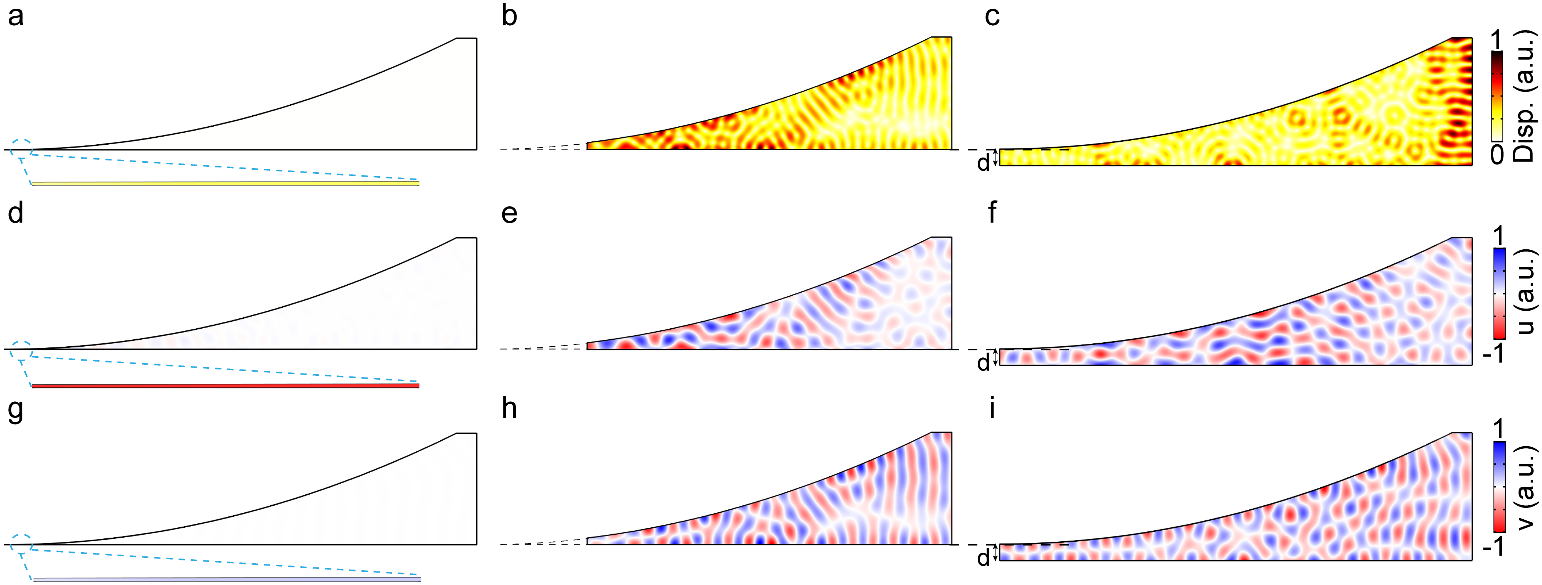


**Fig. S1. Ideal ABH verses practical ABHs. a-c** Distributions for total displacements of an ideal ABH, an intercepted ABH, and a thickened ABH, respectively. **d-f** Distributions for displacements in *x* axis of the ideal ABH, the intercepted ABH, and the thickened ABH, respectively. **g-i** Distributions for displacements in *y* axis of the ideal ABH, the intercepted ABH, and the thickened ABH, respectively. Elastic waves are emitted from the right side.


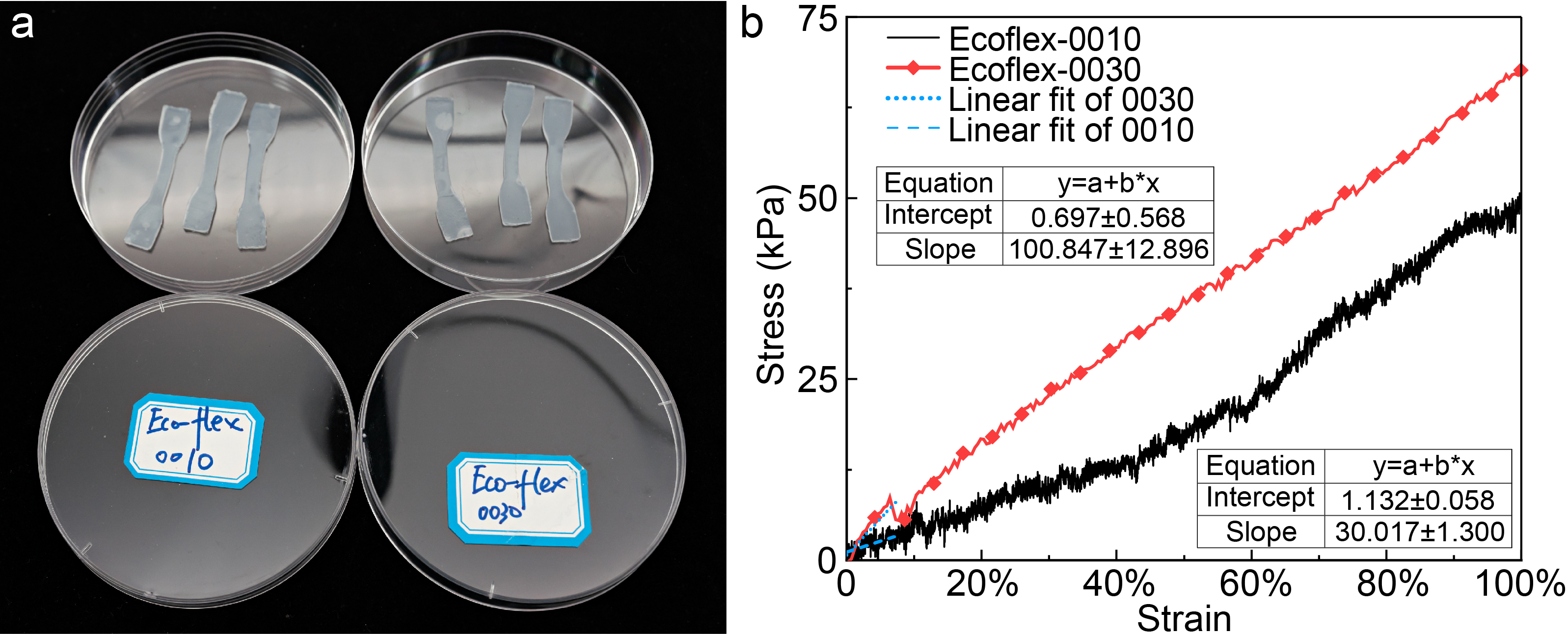


**Fig. S2. Uniaxial tensile tests of Ecoflex-0030 and Ecoflex-0010.** **a** Standard samples of Ecoflex-0030 and Ecoflex-0010. **b** Measured strain-stress curve of Ecoflex-0030 and Ecoflex-0010.


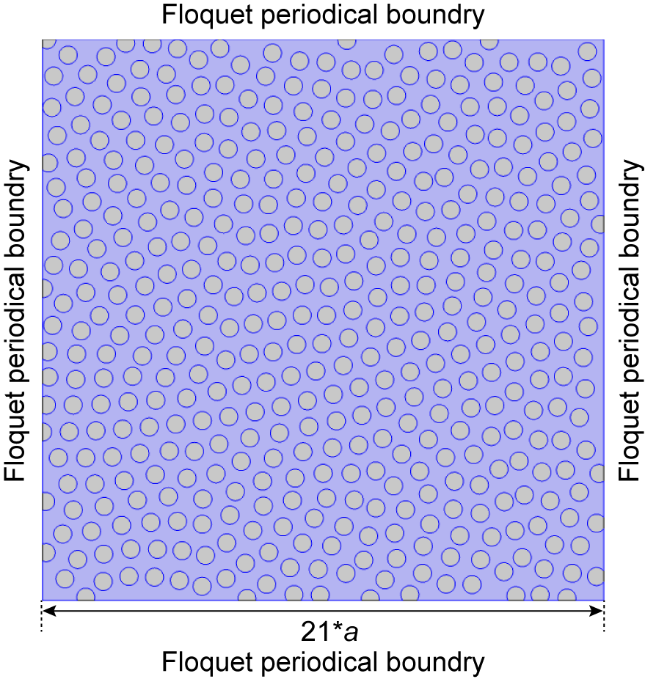


**Fig. S3. Model for calculation of dispersion relationship of DHPS.** The whole square is considered as a single cell. The four borders of the whole square are set to be Floquet periodical boundary.


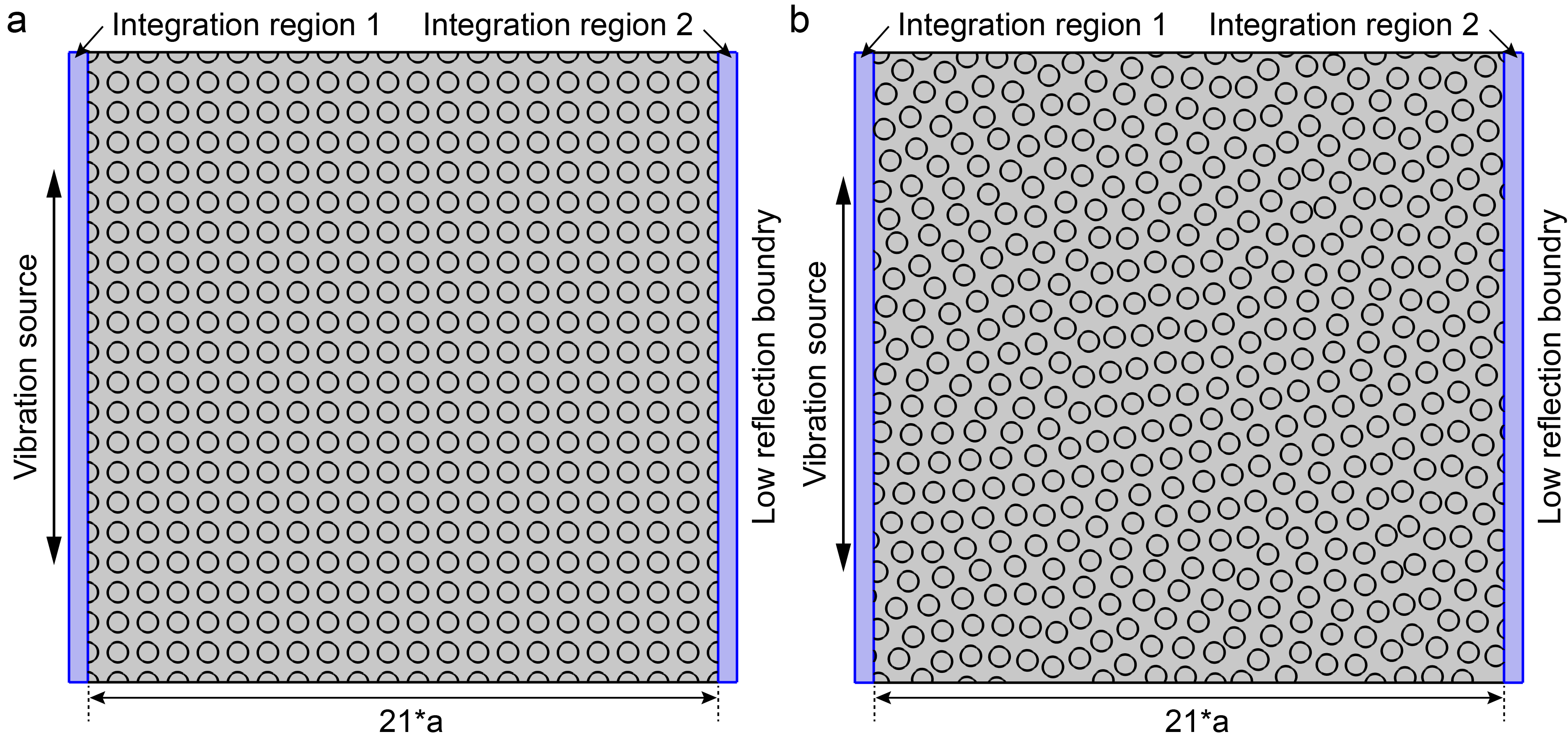


**Fig. S4. Simulation model configurations for transmission of PC (a) and DHPS (b).**


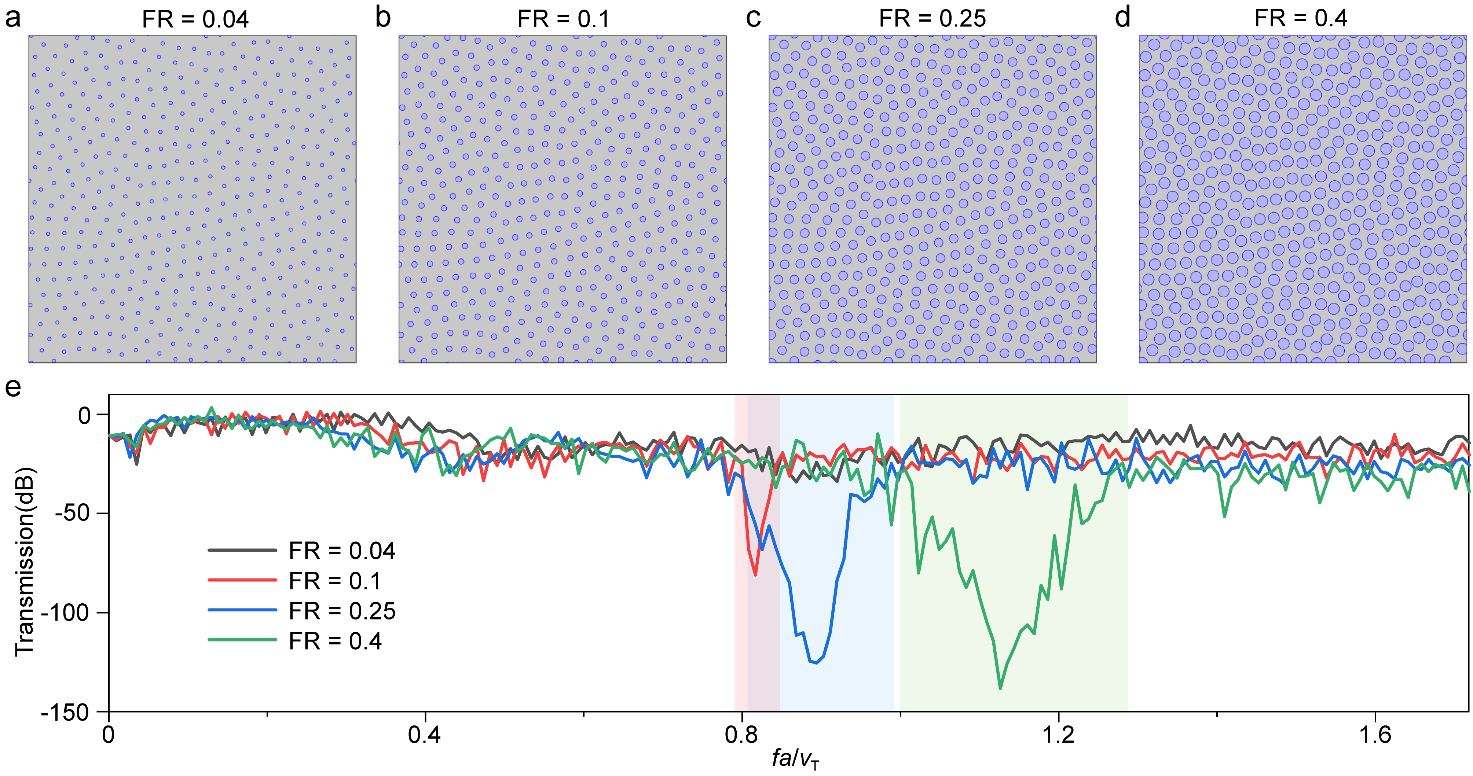


**Fig. S5. The influence of filling ratio (FR) to bandgaps.** **a-d** DHPS model with the filling ratios (by area) being 0.04, 0.1, 0.25, and 0.4, respectively. **e** Transmission of the four configurations in a-d. bandgap regions are highlighted with different color block.


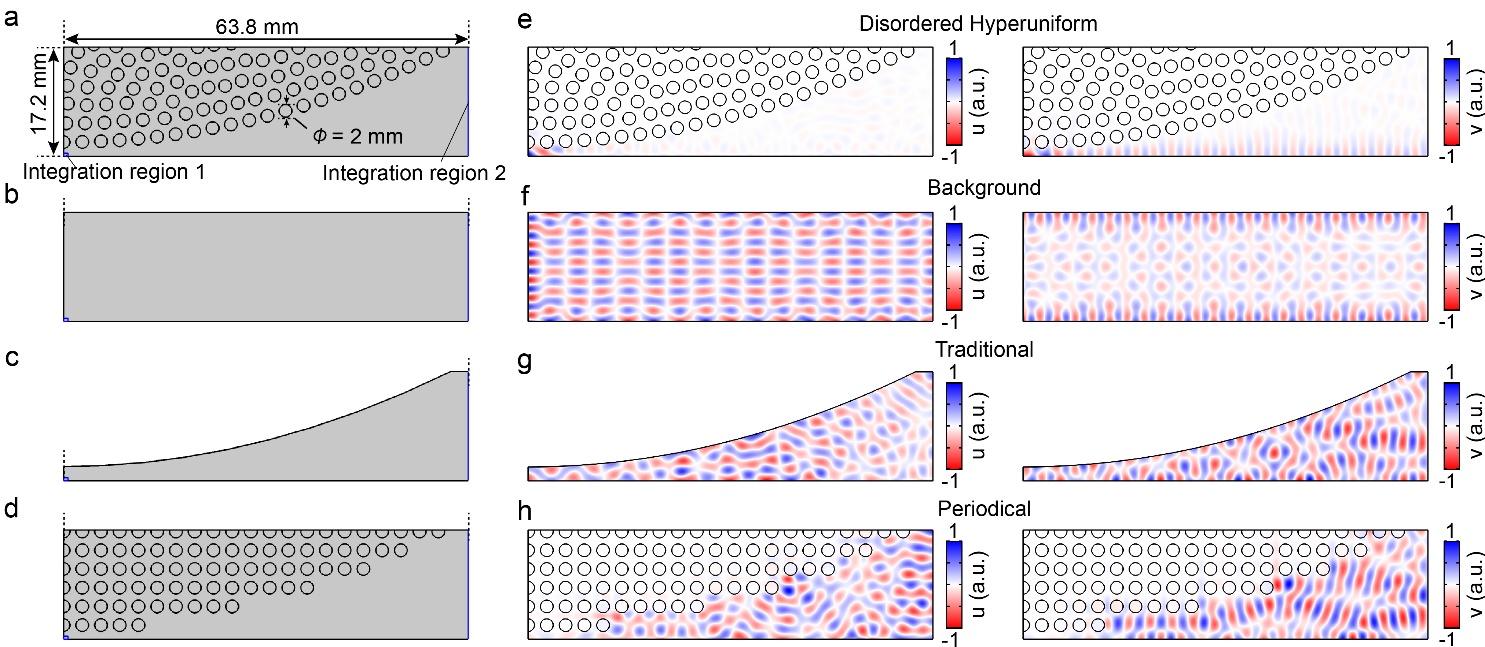


**Fig. S6. Sketches of different configurations in simulation.** **a** Sketch of the DHEM design. Blue blocks indicate the integration regions for enhancement calculation. The sizes for integration region 1 and 2 are 0.7 mm × 0.48 mm and (0.336/17.2) mm × 17.2 mm, respectively. The integration regions and structural parameters for other designs are similar. **b-d** Sketch of the control group, traditional ABH with truncation, and periodical ABH, respectively. **e-h** Vibration distributions when excited from the right sides at *x* direction (**u**) and *y* direction (**v**) for DHEM, the control group, traditional ABH with truncation, and periodical ABH, respectively.


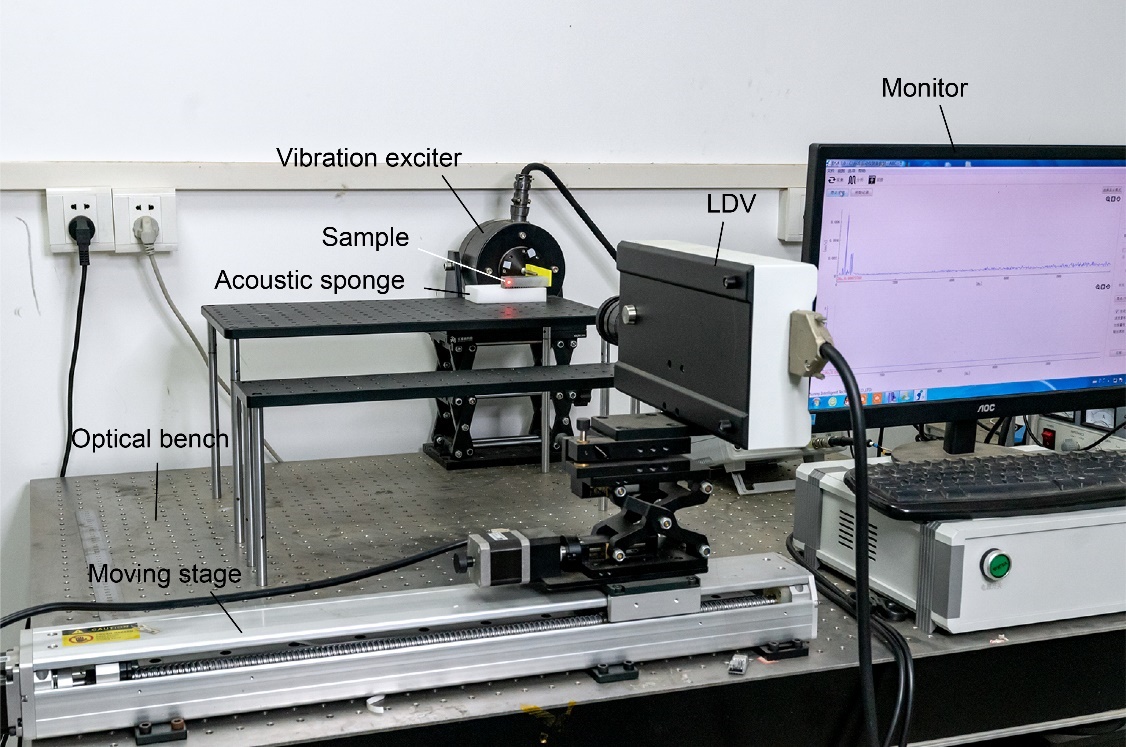


**Fig. S7. Experimental setup for measuring concentration effect of DHEM.**


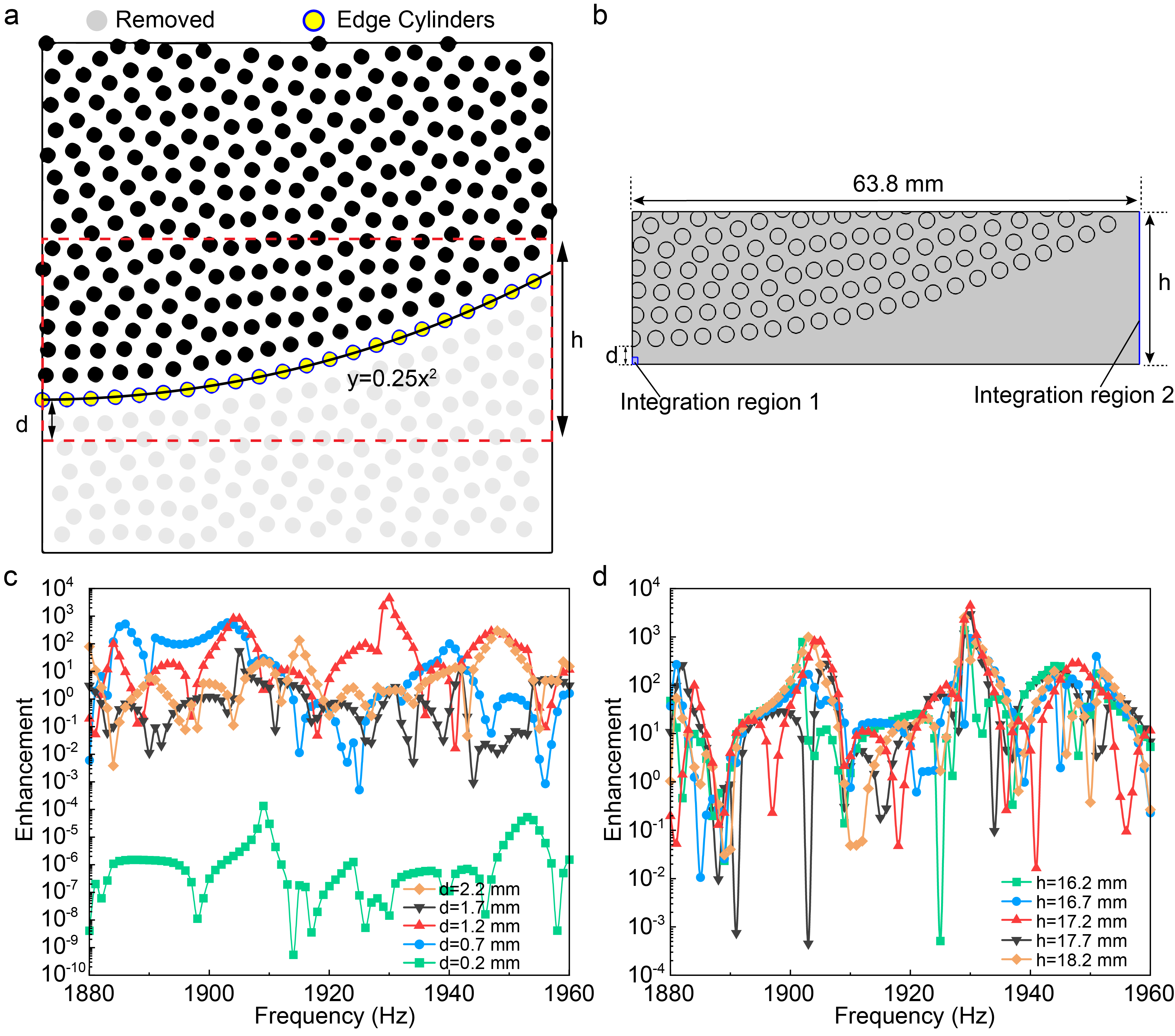


**Fig. S8. Optimization of structural parameters of DHEM.** **a** Schematic for extracting the objective pattern from the original DH pattern. **b** Sketch that indicates the parameters need to be optimized. **c** Enhancement profiles with different bottom thickness *d* in a broad frequency range. **d** Enhancement profiles with different height *h* in a broad frequency range.


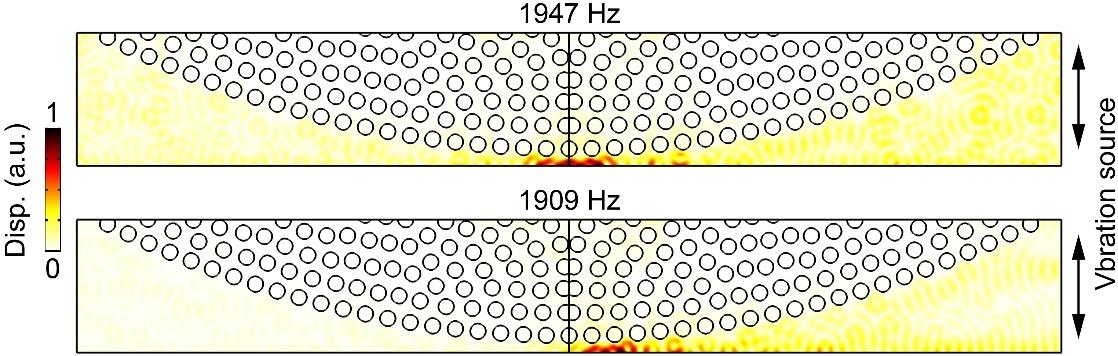


**Fig. S9. Mirror model to verify the design of DHEM at two typical frequencies: 1909 Hz and 1947 Hz.**


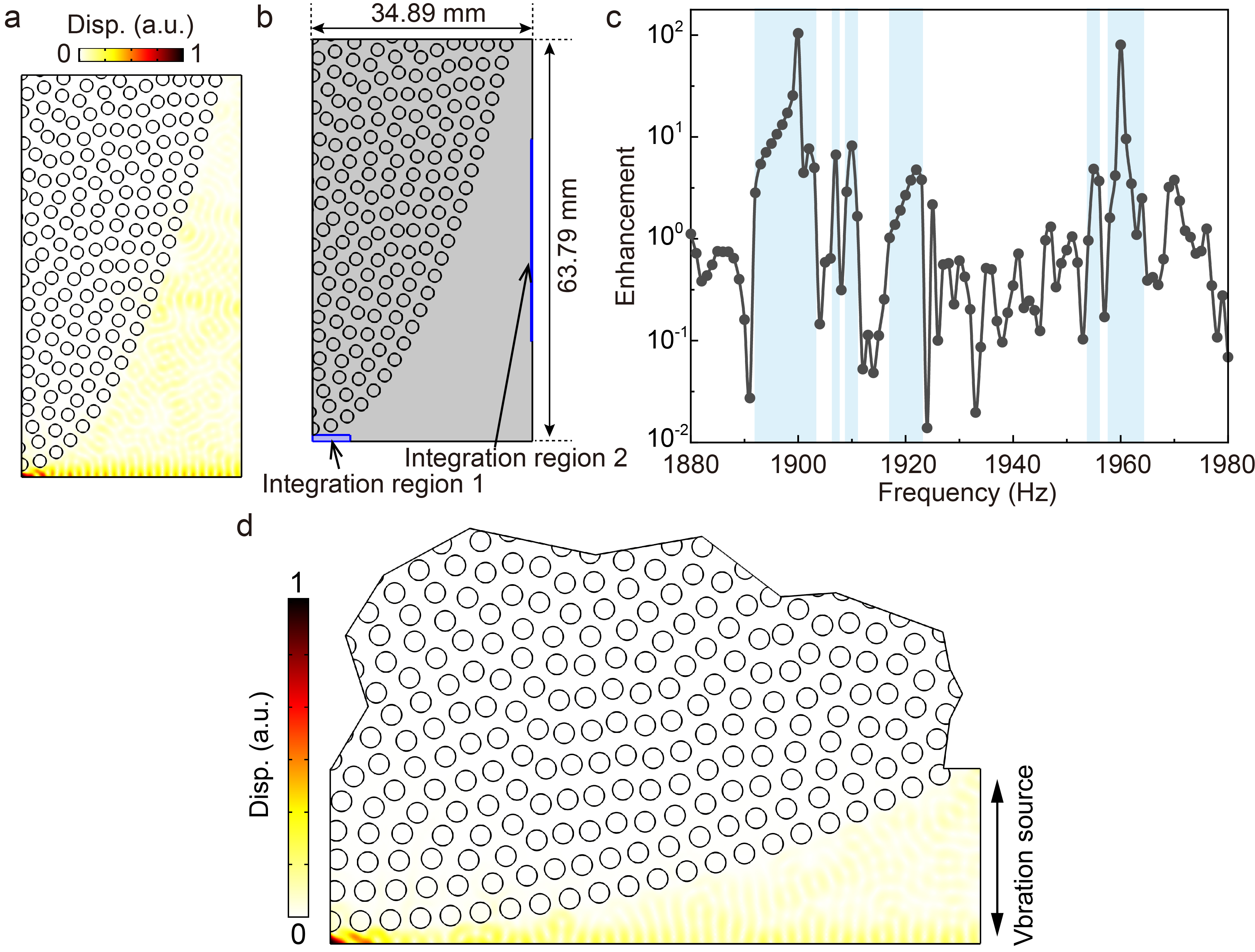


**Fig. S10. The universality of the DHEM method.** **a** simulated total displacement distribution of the DHEM model with steep power-law profile when excited from the right side. **b** Sketch of the steeper DHEM design. Blue blocks indicate the integration regions for enhancement calculation. The sizes for integration region 1 and 2 are 6mm × 1 mm and 0.25 mm × 32 mm, respectively. **c** Enhancement of steeper DHEM in a broad frequency range. **d** DHEM with an amorphous upper border.


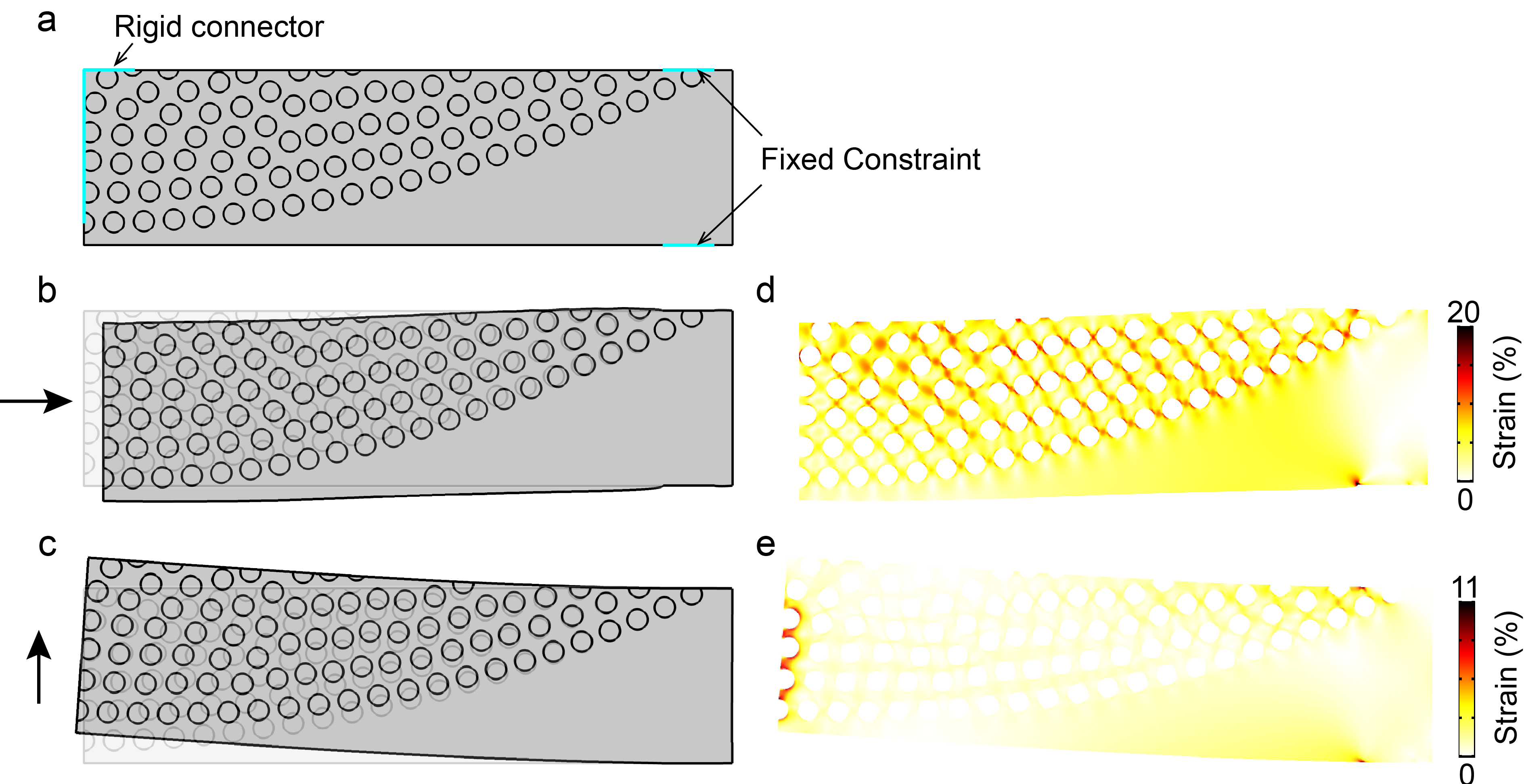


**Fig. S11. Schematic of deformation process.** **a** Boundary condition applied in deformation simulation. **b** Compressing deformation. The DHEM was compressed by 2 mm in x direction. **c** Bending deformation. The DHEM was stretched 3mm upwards at the rigid connector when the right part was fixed. **d**, **e** Stain distributions of **b**, **c**, respectively. ­­­


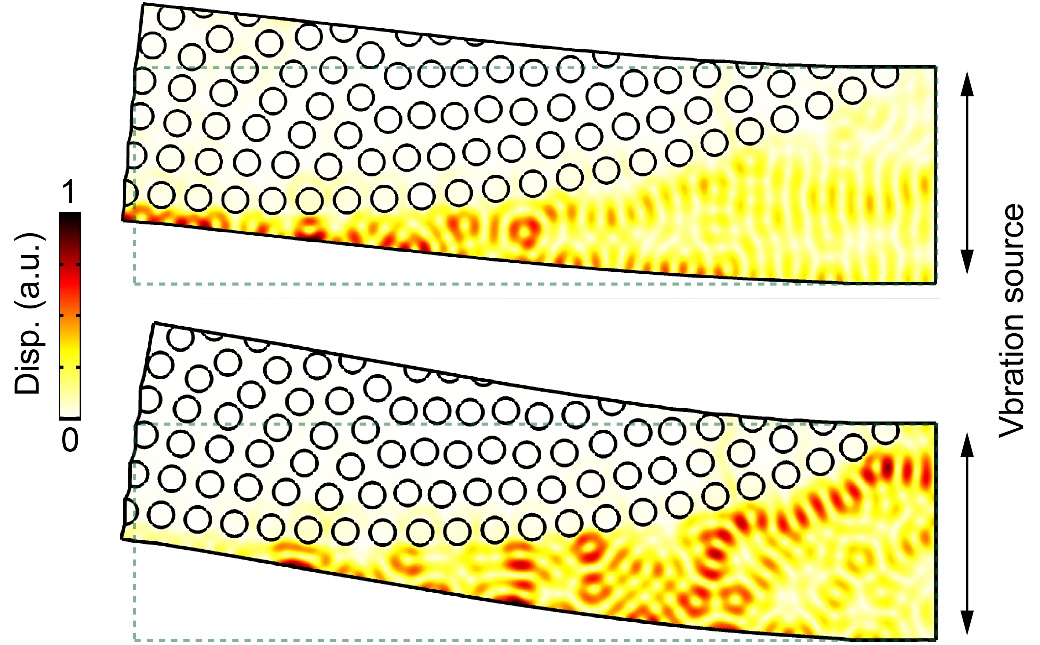


**Fig. S12. Vibration distributions of DHEMs under large bending deformation when excited from the right side at 1930 Hz.** The upper model was pulled up the left side by 5 mm. The lower model was pulled up the left side by 8 mm. Green dash boxes in the figure indicate the origin shape of DHEMs. The concentration effect fades away when the bending strain gets larger.


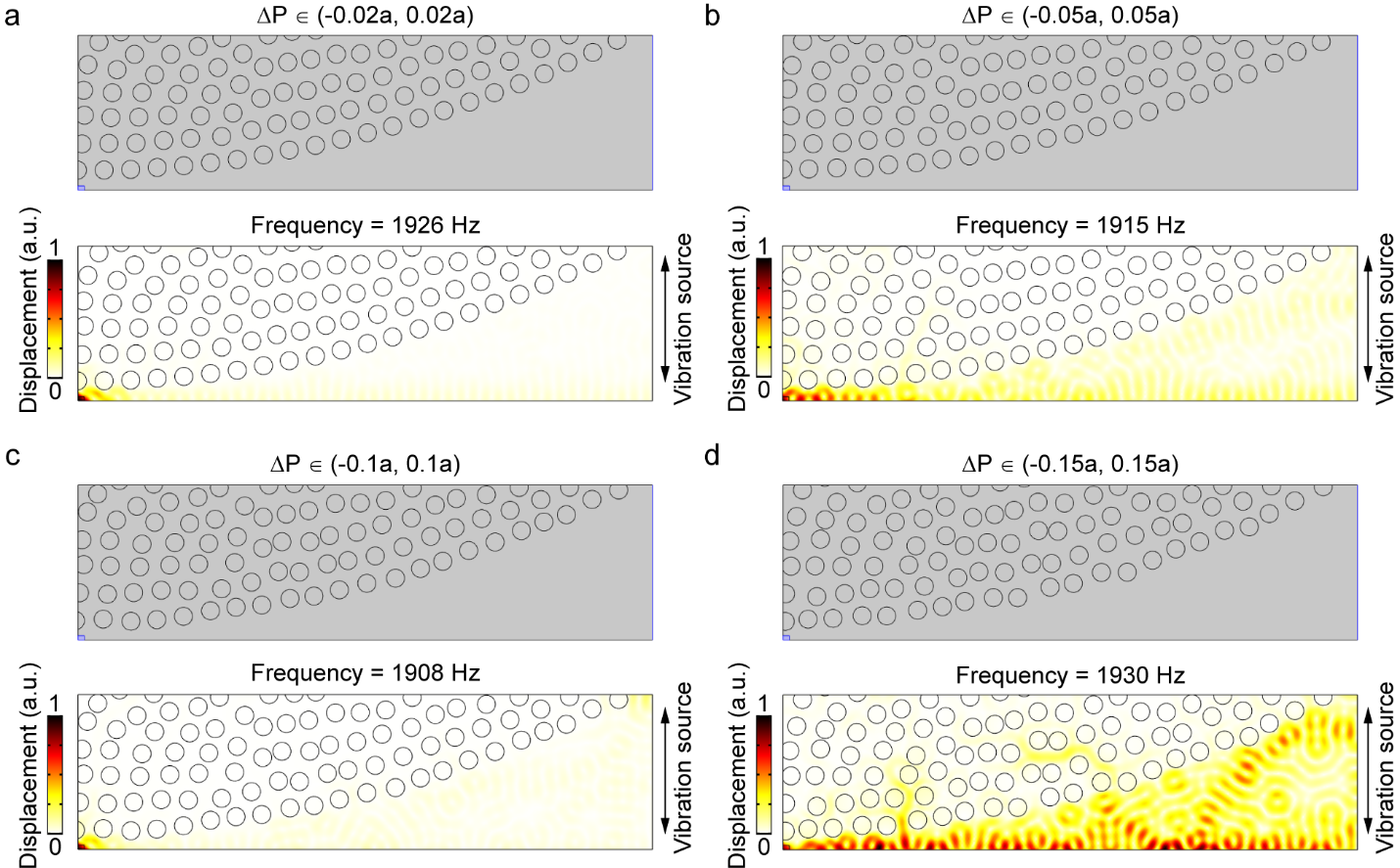


**Fig. S13. Simulated concentration effects of DHEM with position deviation.** **a-d** Sketches of the DHEM design (upper panel) and corresponding simulated vibration distributions (lower panel) at Δ*P*∈(-0.02*a*, 0.02*a*), Δ*P*∈(-0.05*a*, 0.05*a*), Δ*P*∈(-0.1*a*, 0.1*a*), Δ*P*∈(-0.15*a*, 0.15*a*), respectively.


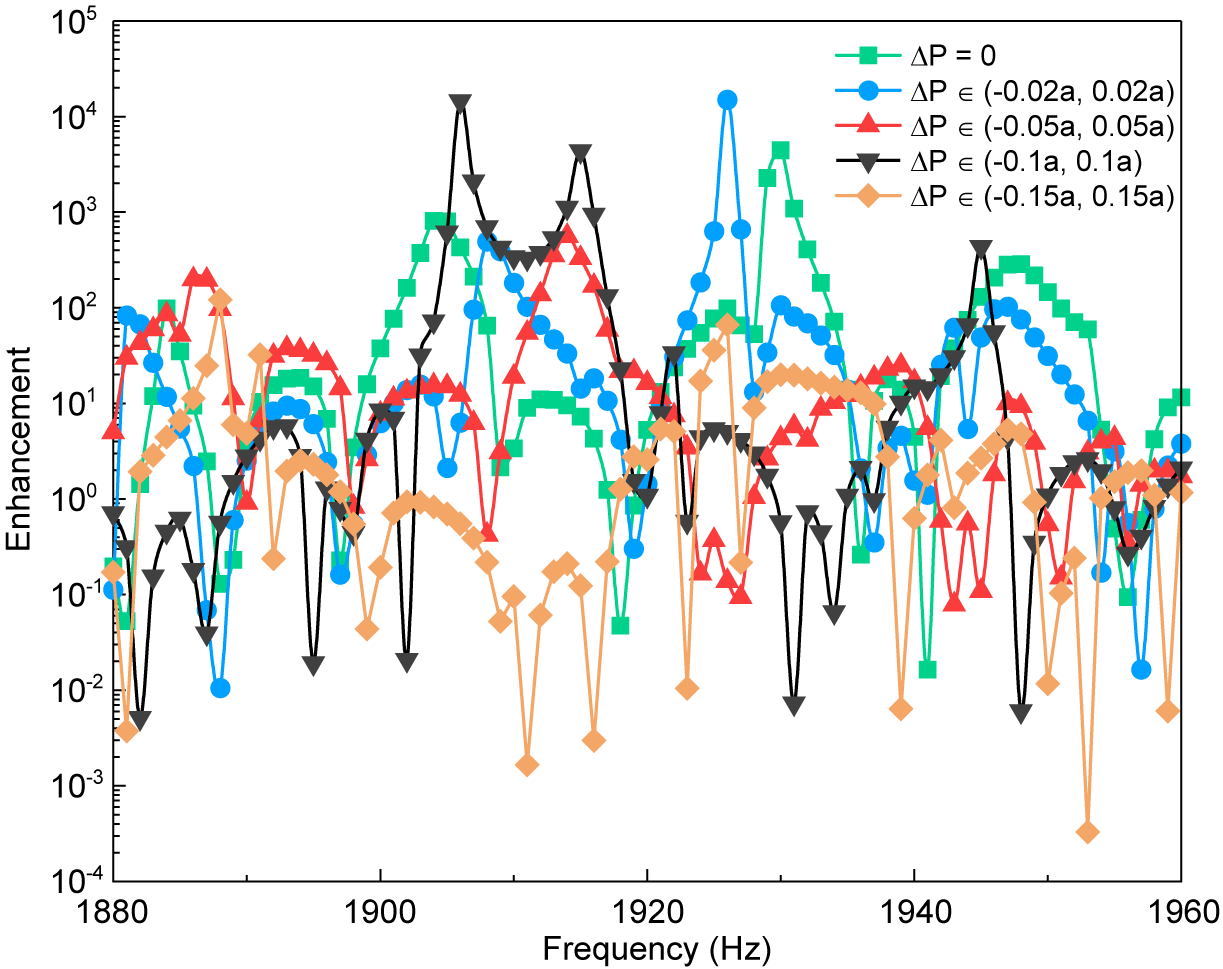


**Fig. S14. Enhancement factor among 1880 Hz to 1960 Hz of the DHEM with Δ*P* = 0, Δ*P*∈(-0.02*a*, 0.02*a*), Δ*P*∈(-0.05*a*, 0.05*a*), Δ*P*∈(-0.1*a*, 0.1*a*), Δ*P*∈(-0.15*a*, 0.15*a*).**


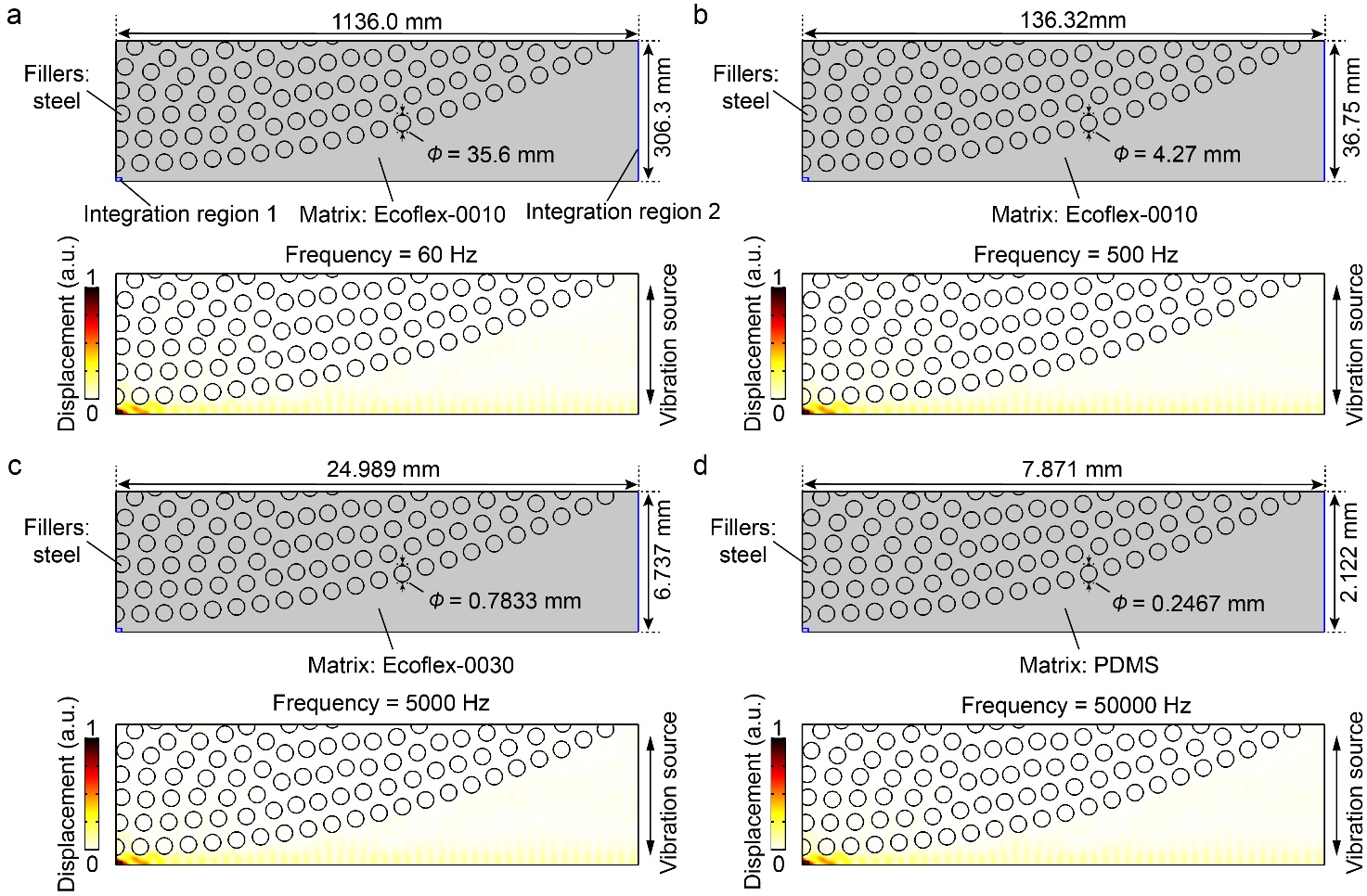


**Fig. S15. Vibration concentration models at broad frequencies.** **a-d** Sketches of the DHEM design and corresponding simulated vibration distributions at 60 Hz, 500 Hz, 5000 Hz, and 50000 Hz, respectively. The enhancements for 60 Hz, 500 Hz, 5000 Hz, and 50000 Hz are 5070 times, 5950 times, 5830times, and 5530 times, respectively.

Table S1. Summary and comparison of various mechanism of vibration harvesting.

| Mechanism | Source | Enhancement | Working frequency | Deformability | Size | Examples |
| --- | --- | --- | --- | --- | --- | --- |
| Phononic crystals | Vibration | **~**20 times | 30 kHz~70 kHz | None | >20 cm × 20 cm | *Nano Energy* 57, 327 (2019)  *Appl. Phys. Lett.* 109, 063902 (2016) |
| Triboelectric nanogenerator with structures | Vibration | 30 times**~**100 times | 10 Hz~60 Hz | None | >6 cm × 5 cm | *Adv. Energy Mater.* 9, 1902460 (2019)  *Adv. Energy Mater.* 9, 1902824 (2019) |
| magneto-mechano-harvester | Magneto-vibration | **~**100 times | ~60 Hz | None | >10 cm × 10 cm | *Energy Environ. Sci.* 13, 4238 (2020)  *Energy Environ. Sci.* 13, 1462 (2020) |
| DHEM | Vibration | **~4000** **times** | **100 Hz~10 kHz** | **Stretchable and bendable** | **>1 cm** × **5 cm** | **This work** |
